# Association of *PHACTR1* with Coronary Artery Calcium Differs by Sex and Cigarette Smoking

**DOI:** 10.3390/jcdd11070194

**Published:** 2024-06-27

**Authors:** Kirsten Voorhies, Kendra Young, Fang-Chi Hsu, Nicholette D. Palmer, Merry-Lynn N. McDonald, Sanghun Lee, Georg Hahn, Julian Hecker, Dmitry Prokopenko, Ann Chen Wu, Elizabeth A. Regan, Dawn DeMeo, Greg L. Kinney, James D. Crapo, Michael H. Cho, Edwin K. Silverman, Christoph Lange, Matthew J. Budoff, John E. Hokanson, Sharon M. Lutz

**Affiliations:** 1 Department of Population Medicine, Harvard Pilgrim Health Care Institute, Boston, MA 02115, USA; 2 Department of Epidemiology, University of Colorado Anschutz Medical Campus, Aurora, CO 80045, USA; 3 Department of Biostatistics and Data Science, Division of Public Health Sciences, Wake Forest University School of Medicine, Winston-Salem, NC 27101, USA; 4 Department of Biochemistry, Wake Forest University School of Medicine, Winston-Salem, NC 27101, USA; 5 Division of Pulmonary, Allergy and Critical Care Medicine, Department of Medicine, University of Alabama at Birmingham, Birmingham, AL 35212, USA; 6 Department of Genetics, University of Alabama at Birmingham, Birmingham, AL 35233, USA; 7 Division of Medicine, Department of Medical Consilience, Graduate School, Dankook University, Yongin 16890, Republic of Korea; 8 Brigham and Women’s Hospital, Division of Pharmacoepidemiology and Pharmacoeconomics, and Department of Medicine, Harvard Medical School, Boston, MA 02120, USA; 9 Channing Division of Network Medicine, Brigham and Women’s Hospital and Harvard Medical School, Boston, MA 02115, USA; 10 Genetics and Aging Research Unit and the McCance Center for Brain Health, Department of Neurology, Massachusetts General Hospital, Boston, MA 02114, USA; 11 Department of Medicine, National Jewish Health, Denver, CO 80206, USA; 12 Division of Pulmonary and Critical Care Medicine, Brigham and Women’s Hospital, Harvard Medical School, Boston, MA 02115, USA; 13 Department of Biostatistics, Harvard T.H. Chan School of Public Health, Boston, MA 02115, USA; 14 Lundquist Institute at Harbor-UCLA Medical Center, Torrance, CA 90502, USA

**Keywords:** coronary artery calcium, *PHACTR1*, *CDKN2B-AS1*

## Abstract

**Background:** Coronary artery calcium (CAC) is a marker of subclinical atherosclerosis and is a complex heritable trait with both genetic and environmental risk factors, including sex and smoking. **Methods:** We performed genome-wide association (GWA) analyses for CAC among all participants and stratified by sex in the COPDGene study (*n* = 6144 participants of European ancestry and *n* = 2589 participants of African ancestry) with replication in the Diabetes Heart Study (DHS). We adjusted for age, sex, current smoking status, BMI, diabetes, self-reported high blood pressure, self-reported high cholesterol, and genetic ancestry (as summarized by principal components computed within each racial group). For the significant signals from the GWA analyses, we examined the single nucleotide polymorphism (SNP) by sex interactions, stratified by smoking status (current vs. former), and tested for a SNP by smoking status interaction on CAC. **Results:** We identified genome-wide significant associations for CAC in the chromosome 9p21 region [*CDKN2B-AS1*] among all COPDGene participants (*p* = 7.1 × 10^−14^) and among males (*p* = 1.0 × 10^−9^), but the signal was not genome-wide significant among females (*p* = 6.4 × 10^−6^). For the sex stratified GWA analyses among females, the chromosome 6p24 region [*PHACTR1*] had a genome-wide significant association (*p* = 4.4 × 10^−8^) with CAC, but this signal was not genome-wide significant among all COPDGene participants (*p* = 1.7 × 10^−7^) or males (*p* = 0.03). There was a significant interaction for the SNP rs9349379 in *PHACTR1* with sex (*p* = 0.02), but the interaction was not significant for the SNP rs10757272 in *CDKN2B-AS1* with sex (*p* = 0.21). In addition, *PHACTR1* had a stronger association with CAC among current smokers (*p* = 6.2 × 10^−7^) than former smokers (*p* = 7.5 × 10^−3^) and the SNP by smoking status interaction was marginally significant (*p* = 0.03). *CDKN2B-AS1* had a strong association with CAC among both former (*p* = 7.7 × 10^−8^) and current smokers (*p =* 1.7 × 10^−7^) and the SNP by smoking status interaction was not significant (*p =* 0.40). **Conclusions:** Among current and former smokers of European ancestry in the COPDGene study, we identified a genome-wide significant association in the chromosome 6p24 region [*PHACTR1*] with CAC among females, but not among males. This region had a significant SNP by sex and SNP by smoking interaction on CAC.

## 1. Introduction

Coronary artery calcium (CAC), as measured by computed tomography (CT), is a marker of subclinical atherosclerosis. CAC has been shown to strongly correlate with the amount of atherosclerotic plaque and can identify asymptomatic individuals who are at risk for myocardial ischemia (MI) [1,2]. The extent and severity of CAC also subsequently predict future risk of coronary disease events such as MI or angina [3].

CAC is a complex heritable trait with both genetic and environmental risk factors, including sex and cigarette smoking. In Americans of European ancestry, quantitative measures of CAC have an estimated heritability between 40 and 60% [4,5]. Through genome-wide association (GWA) studies, 12 regions have been associated with CAC [4,5,6,7,8,9] on chromosomes 2 (Apolipoprotein B [*APOB*]), 6 (Phosphatase And Actin Regulator 1 [*PHACTR1*], MicroRNA 548h-5/Ectonucleotide Pyrophosphatase/Phosphodiesterase 3 [*miR-548h-5*/*ENPP3*]), 7 (Insulin-Like Growth Factor Binding Protein 3 [*IGFBP3*]), 9 (CDKN2B Antisense RNA 1 [*CDKN2B-AS1*]), 10 ([*AL512640.1*], C-X-C Motif Chemokine Ligand 12 [*CXCL12*], AT-Rich Interaction Domain 5B [*ARID5B*], Adenosine Kinase [*ADK*]), 12 (Fibroblast Growth Factor 23 [*FGF23*]), 13 (Collagen Type IV Alpha 1 Chain/Collagen Type IV Alpha 2 Chain [*COL4A1*/*COL4A2*]), 15 (ADAM Metallopeptidase With Thrombospondin Type 1 Motif 7/Mortality Factor 4 Like 1 [*ADAMTS7*/*MORF4L1*]), and 19 (Apolipoprotein E [*APOE*]). Some of these known loci for CAC are pleiotropic and have been associated with other traits. For instance, the 9p21 locus [*CDKN2B-AS1*] has also been associated with a number of cardiovascular manifestations including MI [10], coronary artery disease (CAD) [5,11], risk of abdominal and intracranial aneurysms [12], peripheral arterial disease [12], heart failure [13], sudden cardiac death [14], and stroke [15]. The 6p24 region [*PHACTR1*] has also been associated with CAD, migraine, cervical artery dissection, fibromuscular dysplasia, and hypertension [16,17,18,19].

Some of these known loci for CAC have also been shown to differ by sex [9,20,21]. In a large GWA meta-analysis of CAC comprised of 26,909 individuals of European ancestry and 8867 individuals of African ancestry, sex stratified GWA analyses found genome-wide significant associations (*p* < 5 × 10^−8^) with CAC at *PHACTR1* for both males and females and at *CDKN2B-AS1*/*CDKN2B* for males [9]. In addition, there were two significant single nucleotide polymorphism (SNP) by sex interactions [*ARID5B*, *CDKN2B-AS1*/*CDKN2B*] with a stronger allelic effect in males compared to females despite similar allele frequencies. In a population-based German cohort of 4329 participants for the Heinz Nixdorf Recall Study, sex stratified analyses showed that the chromosome 9p21 [*CDKN2B-AS1*] SNPs (rs1537373 and rs10965219) had a stronger association with CAC in males compared to females and the chromosome 6p24 [*PHACTR1*] SNP (rs9349379) had a stronger association with CAC in females as compared to males [22]. However, only one SNP [rs10965219] in *CDKN2B-AS1* showed a marginally significant SNP by sex interaction after Bonferroni correction (*p =* 0.01) [22].

In addition, smoking is an important environmental risk factor for CAC [23,24,25], and CAC mediates the effect of smoking on cardiovascular disease (CVD) [26]. Nearly one third of the deaths due to CVD are smoking related [27]. However, the role of cigarette smoking on the association of these SNPs with CAC has not been well explored.

In order to examine the genetic susceptibility of CAC among current and former smokers and the role of sex, we performed GWA analyses of CAC among all participants and stratified by sex in the Genetic Epidemiology of COPD (COPDGene) study, a large cohort of current and former smokers enriched with COPD cases [28]. The Diabetes Heart Study (DHS) served as a replication population. In addition, we examined SNP by sex and SNP by smoking status interactions on CAC.

## 2. Materials and Methods

### 2.1. COPDGene Study

The COPDGene study is a multicenter observational study designed to identify and characterize genetic factors associated with COPD and COPD-related phenotypes [28]. This study recruited 10,192 unrelated current and former adult smokers with at least 10 pack-years of smoking history who were of European or African ancestry ages 44 to 81 years. Table 1 details characteristics of the COPDGene participants of European ancestry included in the analyses and Supplement Table S1 details characteristics of the COPDGene participants of African ancestry. We excluded participants with genotyping failure, severe alpha-1 antitrypsin deficiency based on genotyping, or no phenotype data, which resulted in 6144 participants of European ancestry and 2589 participants of African Ancestry. Details of genotyping quality control and imputation have been described previously [29]. All COPDGene participants were genotyped using the Illumina HumanExome arrays (v1.1 and v1.2; Illumina, San Diego, CA, USA).

### 2.2. Diabetes Heart Study (DHS)

DHS is a genetic and epidemiological study of European American (EA) and African American (AA) families with multiple cases of type 2 diabetes (T2D) [30]. Briefly, siblings with T2D and without advanced nephropathy were recruited, and unaffected siblings were also recruited when possible. T2D was defined as diabetes developing after 35 years of age, with initial treatment using a combination of exercise and/or oral agents, not solely insulin, and in the absence of historical evidence of ketoacidosis. The AA-DHS cohort was used to expand DHS and improve the understanding of ancestry-specific differences in the relationship between T2D and associated chronic illnesses through the recruitment of additional unrelated AA participants with T2D [31]. All participants were assessed for measures of subclinical cardiovascular disease [30,31]. Genetic data obtained from the Affymetrix Genome-wide Human SNP Array 5.0 (DHS) and the Illumina 5M array (AA-DHS) were used to capture the replication variants of interest.

### 2.3. CAC Measurement

In the COPDGene study, CAC was measured from high-dose chest computed tomography (CT) scans taken in full inspiration using an established protocol for ungated studies [32]. CAC was classified with a CT threshold of 130 Hounsfield units (HUs) involving three contiguous voxels for identification of a calcific lesion, resulting in a minimum lesion area of 1.02 mm [33]. The lesion score was calculated using the area density method, by multiplying the lesion area by a density factor derived from the maximal Hounsfield unit (HU) within the area as described by Agatston [34,35]. The density factor was assigned in the following manner: 1 for lesions whose maximal density was 130–199 HU, 2 for lesions 200–299 HU, 3 for lesions 300–399 HU, and 4 for lesions > 400 HU. A total coronary artery calcium score was determined by summing individual lesion scores from each of 4 anatomic sites (left main, left anterior descending, circumflex, and right coronary arteries) [32]. Due to the non-normality of the CAC scores, we used a log plus 1 transformation [7,8,33,34,35]. We also analyzed CAC as a binary phenotype (0 for CAC = 0 and 1 for CAC > 0). Similar results were obtained when CAC was analyzed as a binary outcome as compared to the log-transformed continuous outcome; therefore, the results for CAC as a binary outcome are not presented here.

### 2.4. Statistical Methods

GWA analyses were performed in PLINK [36] stratified by race for SNPs with a minor allele frequency greater than 5%. Linear regression analyses of CAC were adjusted for age, sex, current smoking status, BMI, diabetes, self-reported high blood pressure, self-reported high cholesterol, and genetic ancestry (as summarized by principal components computed within each racial group) [37]. In addition, the GWA analyses were stratified by sex. For the GWA analyses among all participants and stratified by sex, a genome-wide significance threshold of 5 × 10^−8^ was used. For SNPs that were genome-wide significant in the GWA analyses, we tested SNP by sex and SNP by smoking status interactions on CAC. For the interaction analyses, the significance level was based on a Bonferroni correction of 0.05/2 = 0.025 for the two SNPs. In addition, we stratified by smoking status (current vs. former smoker) in Table 2 and light (≤10 cigarettes per day), moderate (11–19 cigarettes per day), and heavy (≥20 cigarettes per day) smokers in Supplemental Table S4. For the DHS replication analysis among participants of European ancestry, due to the correlated family structure, generalized estimating equations were used. For the DHS analysis among participants of African ancestry, linear regression models were used. For the DHS analyses, the same covariates listed above were adjusted for.

## 3. Results

### 3.1. Overall and Sex Stratified GWA Analyses

**Figure 1:**
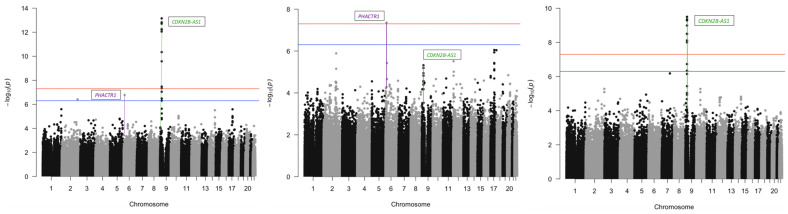
Manhattan plots of GWA analyses of CAC among COPDGene participants of European ancestry and stratified by sex. The plot on the left is for all participants, the middle plot is for females, and the right plot is for males. The red horizontal line represents *p*-values of 5 × 10^−8^ and the blue horizontal line represents *p*-values of 5 × 10^−7^.

Among all COPDGene participants of European ancestry in the overall GWA analysis, multiple SNPs at the chromosome 9p21 region reached genome-wide significance for CAC (*p* = 7.1 × 10^−14^) as seen in Table 2 and the Manhattan plots in Figure 1. For the sex stratified GWA analysis among male COPDGene participants, multiple SNPs at the chromosome 9p21 region reached genome-wide significance (*p* = 1.0 × 10^−9^) for CAC. For the sex stratified GWA analysis among female COPDGene participants, a SNP in the chromosome 6p24 [*PHACTR1*] was genome-wide significant (*p* = 4.4 × 10^−8^). As seen in Supplemental Table S2, there were no genome-wide significant results for CAC among COPDGene participants of African ancestry, possibly due to their smaller sample size (6144 participants of European Ancestry vs. 2589 participants of African ancestry). For DHS, the chromosome 9p21 region [*CDKN2B-AS1*] was marginally significant for CAC among all participants (*p* = 0.02) and males (*p* = 0.01), but not among females (*p* = 0.25). The chromosome 6p24 region [*PHACTR1*] was marginally significant among all participants (*p* = 7.4 × 10^−3^) and females (*p* = 2.5 × 10^−3^), but not among males (*p* = 0.43).

### 3.2. SNP by Sex Interactions

We examined the SNP by sex interactions on CAC for these two signals (*PHACTR1, CDKN2B-AS1*). In the COPDGene study, there was a significant interaction for the SNP in *PHACTR1* with sex on CAC (*p* = 0.02), but the interaction was not significant for the SNP in *CDKN2B-AS1* with sex (*p* = 0.21). The interaction did not replicate within DHS.

### 3.3. The Role of Smoking

For rs9349379 [*PHACTR1*] and rs10757272 [*CDKN2B-AS1*], we examined the association with CAC stratified by smoking status and tested for SNP by smoking status interactions. Among participants of European ancestry in the COPDGene study, rs9349379 [*PHACTR1*] had a stronger association with CAC among current smokers (*p =* 6.2 × 10^−7^) than former smokers (*p =* 7.5 × 10^−3^) and the rs9349379 by smoking status interaction was marginally significant (*p* = 0.03). rs10757272 [*CDKN2B-AS1*] had a strong association with CAC among both former (*p* = 7.7 × 10^−8^) and current smokers (*p =* 1.7 × 10^−7^) and the rs10757272 by smoking status interaction was not significant (*p =* 0.40). While there was a marginally significant association with rs9349379 [*PHACTR1*] among former smokers (*p =* 0.03) in the DHS study, the SNP by smoking status interaction was not significant (*p* = 0.11) and did not replicate.

In addition, we examined the effect of these SNPs on CAC stratified by light, moderate, and heavy smokers in the COPDGene study. While there was a smaller number of light and moderate smokers as compared to heavy smokers as seen in Supplemental Table S3, rs9349379 [*PHACTR1*] had a stronger association with CAC among heavy smokers (*p* = 5.4 × 10^−6^) as compared to moderate (*p* = 0.23) and light smokers (*p* = 4.2 × 10^−3^) among participants of European ancestry as seen in Supplemental Table S4. rs10757272 [*CDKN2B-AS1*] had a stronger association with CAC among heavy smokers (*p* = 8.6 × 10^−11^) as compared to moderate (*p* = 5.6 × 10^−3^) and light smokers (*p* = 7.5 × 10^−3^). However, this trend could be due to the larger number of heavy smokers (*n* = 5242) as compared to moderate (*n* = 459) and light smokers (*n* = 443).

### 3.4. Differences among COPDGene Participants of European and African Ancestry

While there is a strong signal among COPDGene participants of European ancestry for *PHACTR1* and *CDKN2B-AS1*, there is not a strong signal among COPDGene participants of African ancestry for CAC in these regions. However, there are several differences among COPDGene participants of European and African ancestry. Within the COPDGene study, participants of African ancestry had less CAC (41%) compared to participants of European ancestry (65%). However, there was a larger proportion of current smokers among participants of African ancestry (80%) compared to participants of European ancestry (39%). Participants of African ancestry were younger (54.6 years) compared to participants of European ancestry (62.0 years). There is also a significant difference in sample sizes among the participants of European ancestry (*n* = 6144) and participants of African ancestry (*n* = 2589). The allele frequency for the SNPs in *PHACTR1* and *CDKN2B-AS1* was lower among participants of African ancestry (rs9349379 [*PHACTR1*] MAF = 0.08; rs10757272 [*CDKN2B-AS1*] MAF = 0.21) as compared to participants of European ancestry (rs9349379 [*PHACTR1*] MAF = 0.40; rs10757272 [*CDKN2B-AS1*] MAF = 0.50). These factors may have contributed to the differing results between the two populations. 

## 4. Discussion

Among current and former smokers of European ancestry in the COPDGene study, we identified a genome-wide significant association in the chromosome 6p24 region [*PHACTR1*] with CAC among females, but not among males. This SNP rs9349379 in *PHACTR1* had a significant interaction with sex on CAC (*p* = 0.02). This SNP rs9349379 also had a stronger association with CAC among current smokers (*p =* 6.2 × 10^−7^) than former smokers (*p =* 7.5 × 10^−3^) and the SNP by smoking status interaction was marginally significant (*p* = 0.03).

While this SNP rs9349379 in the *PHACTR1* region has been previously associated with CAC [4,9,22] and shown to have a stronger association in females than males [9,22], previous studies examining the SNP by sex interaction in a large meta-analysis including a subset of the COPDGene study [9] and the German cohort for the Heinz Nixdorf Recall Study [22] have not found a significant SNP by sex interaction on CAC (*p* = 0.50 and *p* = 0.37, respectively). Also, these studies have not examined smoking stratified or *PHACTR1* by smoking interactions on CAC. This study demonstrates a significant interaction between rs9349379 and sex on CAC among only current and former smokers of European ancestry. This study also demonstrates a marginally significant interaction between rs9349379 and smoking status on CAC. In addition, this study shows the importance of sex stratified analyses for CAC since the chromosome 6p24 signal was only genome-wide significant among females and not in the overall GWA analysis.

In addition, we identified genome-wide significant associations between SNPs in the chromosome 9p21 region [*CDKN2B-AS1*] among all participants and among males. However, there was not a significant SNP by sex interaction on CAC (*p* = 0.21). *CDKN2B-AS1* had a strong association with CAC among both former and current smokers; however, the SNP by smoking status interaction was not significant (*p =* 0.40). While this region has previously been associated with CAC [4] and shown to have a stronger association in males than females [9,22], this study replicates this association among only current and former smokers.

Note that *PHACTR1* is a protein coding gene on chromosome 6p24 that encodes phosphatase and actin regulator proteins [38]. SNPs in *PHACTR1* have previously been associated with early-onset myocardial infarction and coronary artery disease [39,40]. *CDKN2B-AS1* is a long non-coding RNA gene on chromosome 9p21 that has been found to be associated with age related disease progression; for example, cardiovascular disease [41]. Cardiovascular disease risk alleles from the 9p21 region have been found to be associated with both an increase and decrease in expression of *CDKN2B-AS1* [42,43].

Note that this study had potential limitations. The COPDGene study was ascertained based on smoking status and COPD case-control status. DHS was ascertained for diabetes case-control status. While the DHS study served as a replication population for the COPDGene study, the DHS study contains fewer smokers than the COPDGene study. This may, in part, explain why the smoking interaction with the SNP rs9349379 in *PHACTR1* did not replicate in the DHS. This study also failed to identify any genome-wide signals for CAC among participants of African ancestry. While COPDGene included a substantial number of participants of African ancestry, the sample was considerably smaller than that of participants of European ancestry. In addition, the prevalence of CAC is less among participants of African ancestry compared to participants of European ancestry in the COPDGene study. The null results in participants of African ancestry may indicate a true underlying difference in the genetic susceptibility of CAC in participants of African ancestry compared to European ancestry or may reflect less statistical power due to the smaller sample size and less prevalence of CAC.

## Figures and Tables

**Table 1:** Characteristics of COPDGene and DHS participants of European ancestry included in the GWA analysis. Medical conditions are by patient self-report. For continuous variables, the mean is given first, followed by the standard deviation in parentheses.

	COPDGene	DHS
Sample size	6144	1115
Sex (% male)	52.4%	46.6%
Age (years)	62.0 (8.8)	62.3 (9.3)
BMI (kg/m^2^)	28.7 (6.0)	31.8 (6.5)
Diabetes mellitus (%)	12.1%	83.9%
High blood pressure (%)	42.2%	85.9%
High cholesterol (%)	45.0%	49.2%
Current smoking (%)	39.2%	17.0%
Former smoking (%)	60.8%	42.0%
Never smoking (%)	-	41.0%
CAC (mean [min–max])	202.1 [0–4970]	168.2 [0–5041.5]
CAC > 0 (%)	64.6%	92.5%

**Table 2:** Genome-wide significant results for CAC in the COPDGene study with replication in the DHS study for participants of European ancestry. For the GWA analyses, all *p*-values less than 5 × 10^−8^ are in green, and yellow highlighted cells are marginally significant with 5 × 10^−8^ < *p* < 0.05. For the interactions and smoking status analyses, the significance level was based on a Bonferroni correction of 0.05/2 = 0.025 for the 2 SNPs. As a result, the *p*-values less than 0.025 are in green, and yellow highlighted cells are marginally significant with 0.025 < *p* < 0.05. Base pairs are based on build GR38.

SNP/CHR/Base Pair/Gene/Coded Allele	Study	All			Male			Female			SNP by Sex Interaction			Former Smokers			Current Smokers			SNP by Smoking Status Interaction		
		β	SE	*p*	β	SE	*p*	β	SE	*p*	β	SE	*p*	β	SE	*p*	β	SE	*p*	β	SE	*p*
rs9349379 6 12903725 *PHACTR1* *G*	COPDGene	0.22	0.04	1.7 × 10^−7^	0.13	0.06	0.03	0.32	0.06	4.4 × 10^−8^	0.19	0.08	0.02	0.15	0.05	7.5 × 10^−3^	0.34	0.07	6.2 × 10^−7^	0.19	0.09	0.03
	DHS	0.26	0.10	7.4 × 10^−3^	0.10	0.13	0.43	0.45	0.15	2.5 × 10^−3^	0.30	0.19	0.11	0.31	0.15	0.03	0.12	0.24	0.61	−0.45	0.28	0.11
rs10757272 9 22088261 *CDKN2B-AS1* *T*	COPDGene	0.31	0.04	7.1 × 10^−14^	0.36	0.06	1.0 × 10^−9^	0.26	0.06	6.4 × 10^−6^	−0.10	0.08	0.21	0.28	0.05	7.7 × 10^−8^	0.34	0.07	1.7 × 10^−7^	0.07	0.08	0.40
	DHS	0.22	0.09	0.02	0.29	0.11	0.01	0.17	0.15	0.25	−0.11	0.17	0.51	0.15	0.14	0.28	0.40	0.24	0.09	0.14	0.25	0.58

## Data Availability

The datasets used in this paper can be found at dbGaP https://www.ncbi.nlm.nih.gov/projects/gap/cgi-bin/study.cgi?study_id=phs000179.v1.p1 (accessed on 14 March 2024).

## Supplementary Materials

The following supporting information can be downloaded at: https://www.mdpi.com/article/10.3390/jcdd11070194/s1, Supplemental Table S1. Characteristics of COPDGene and Diabetes Heart Study (DHS) participants of African ancestry. Supplemental Table S2. Results for the GWA analyses of CAC in COPDGene with replication in DHS for participants of African ancestry. Supplemental Table S3. Number of COPDGene participants of European and African ancestry by light, moderate, and heavy smokers. Supplemental Table S4. Results for the COPDGene study stratifying by light (≤10 cigarettes per day), moderate (11–19 cigarettes per day), and heavy (≥20 cigarettes per day) smokers. COPDGene Phase 3 Additional Acknowledgments.
