# Supplementary material for: Association of PHACTR1 with Coronary Artery Calcium Differs by Sex and Cigarette Smoking
Source: J Cardiovasc Dev Dis. 2024 Jun 27;11(7):194. doi: 10.3390/jcdd11070194 (PMC11276683; doi:10.3390/jcdd11070194)
Supplement: Supplementary file 1 [file jcdd-11-00194-s001.zip › jcdd-3058437-supplementary.pdf]

Supplemental Materials: Association of *PHACTR1* with coronary artery calcium differs by sex and cigarette smoking

Kirsten Voorhies[1], Kendra Young[2], Fang-Chi Hsu[3], Nicholette D. Palmer[4], Merry-Lynn N. McDonald[5,6], Sanghun Lee[7], Georg Hahn[8], Julian Hecker[9], Dmitry Prokopenko[10], Ann Chen Wu[1], Elizabeth A. Regan[11], Dawn DeMeo[9,12], Greg L. Kinney[2], James D. Crapo[11], Michael H. Cho[9,12], Edwin K. Silverman[9,12], Christoph Lange[13], Matthew J. Budoff[14], John E. Hokanson[2], Sharon M. Lutz[1,13,*]

1 Department of Population Medicine, Harvard Pilgrim Health Care Institute, Boston, MA, USA

2 Department of Epidemiology, University of Colorado Anschutz Medical Campus, Aurora, Colorado, USA

3 Department of Biostatistics and Data Science, Division of Public Health Sciences, Wake Forest University School of Medicine, Winston-Salem, NC, USA

4 Department of Biochemistry, Wake Forest University School of Medicine, Winston-Salem, NC, USA

5 Division of Pulmonary, Allergy and Critical Care Medicine, Department of Medicine, University of Alabama at Birmingham, Birmingham, AL, USA

6 Department of Genetics, University of Alabama at Birmingham, Birmingham, AL, USA

7 Division of Medicine, Department of Medical Consilience, Graduate School, Dankook University, Yongin 16890, Republic of Korea

8 Brigham and Women's Hospital, Division of Pharmacoepidemiology and Pharmacoeconomics, and Department of Medicine, Harvard Medical School, Boston, MA, USA

9 Channing Division of Network Medicine, Brigham and Women's Hospital and Harvard Medical School, Boston, Massachusetts, MA, USA

10 Genetics and Aging Research Unit and the McCance Center for Brain Health, Department of Neurology, Massachusetts General Hospital, Boston, MA, USA

11 Department of Medicine, National Jewish Health, Denver, Colorado, USA

12 Division of Pulmonary and Critical Care Medicine, Brigham and Women's Hospital, Harvard Medical School, Boston, Massachusetts, USA

13 Department of Biostatistics, Harvard T.H. Chan School of Public Health, Boston, MA, USA

14 Lundquist Institute at Harbor-UCLA Medical Center, Torrance, CA, USA

Supplemental Table S1. Characteristics of COPDGene and Diabetes Heart Study (DHS) participants of African ancestry included in genome-wide association analysis and replication. For continuous variables, the mean is given first, followed by the standard deviation.

	**COPDGene**	**DHS**
Sample size	2,589	751
Sex (% male)	55.9%	40.0%
Age (years)	54.6 (7.2)	56.5 (9.5)
BMI (kg/m^2)	29.1 (6.7)	34.9 (8.2)
Diabetes mellitus (%)	15.0%	96.8%
High blood pressure (%)	45.1%	96.8%
High cholesterol (%)	25.2%	96.8%
Current smoking (%)	80.1%	22.9%
Never smoking (%)	-	40.6%
CAC (mean [min-max])	79.5 [0-2389]	747.5 [0-11845]
CAC >0 (%)	40.7%	79.5%

Supplemental Table S2. Results for the GWA analyss of CAC in COPDGene with replication in DHS for participants of African ancestry among all participants and stratified by sex and smoking status. Note: Yellow highlighted cells are marginally significant with $5x10^{-8} < P < 0.05$.

SNP/ CHR/ Gene/ Coded Allele	Study	All			Male			Female			SNP by Sex Interaction			Former Smokers			Current Smokers			SNP by Smoking Status Interaction		
		β	SE	P	β	SE	P	β	SE	P	β	SE	P	β	SE	P	β	SE	P	β	SE	P
rs9349379 6 *PHACTR1* *G*	COPDGene	0.26	0.11	0.02	0.34	0.15	0.03	0.15	0.16	0.34	-0.15	0.22	0.48	0.26	0.25	0.30	0.25	0.12	0.05	-0.10	0.26	0.71
	Diabetes Heart Study	0.53	0.24	0.03	0.80	0.39	0.04	0.36	0.30	0.24	-0.59	0.45	0.20	0.96	0.46	0.04	0.08	0.47	0.87	-0.48	0.56	0.40
rs10757272 9 *CDKN2B-AS1* *T*	COPDGene	$-9.5x10^{-3}$	0.07	0.90	0.03	0.10	0.77	-0.05	0.10	0.61	-0.09	0.14	0.53	0.14	0.18	0.43	-0.04	0.08	0.57	-0.17	0.18	0.34
	Diabetes Heart Study	0.04	0.17	0.80	0.40	0.29	0.17	-0.20	0.21	0.35	-0.66	0.35	0.06	0.24	0.32	0.45	0.32	0.36	0.38	0.28	0.43	0.51

Supplemental Table S3. Number of COPDGene participants of European ancestry and African ancestry by smoking status (light smokers, moderate smokers, and heavy smokers).

Ancestry	Light smokers (<=10 cigarettes per day)	Moderate smokers (11-19 cigarettes per day)	Heavy smokers (>= 20 cigarettes per day)
European ancestry	443	459	5242
African ancestry	496	347	1746

Supplemental Table S4. Results for the COPDGene study stratifying by light (<=10 cigarettes per day), moderate (11-19 cigarettes per day), and heavy (>= 20 cigarettes per day) smokers. The significance level was based on a Bonferroni correction of 0.05/2=0.025. As a result, the p-values less than 0.025 are in green.

SNP/ CHR/ Gene/ Coded Allele	Ancestry	Light smokers			Moderate Smokers			Heavy Smokers		
		β	SE	P	β	SE	P	β	SE	P
rs9349379<br>6	European ancestry	0.45	0.16	4.2E-3	0.18	0.15	0.23	0.21	0.05	5.4E-6
PHACTR1<br>G	African ancestry	0.27	0.24	0.26	0.40	0.30	0.18	0.21	0.14	0.12
rs10757272<br>9	European ancestry	0.42	0.16	7.5E-3	0.42	0.15	5.6E-3	0.29	0.04	8.6E-11
CDKN2B-AS1<br>T	African ancestry	0.19	0.16	0.24	-0.03	0.19	0.87	-0.07	0.09	0.40

COPDGene Phase 3

Grant Support and Disclaimer

The project described was supported by Award Number U01 HL089897 and Award Number U01 HL089856 from the National Heart, Lung, and Blood Institute. The content is solely the responsibility of the authors and does not necessarily represent the official views of the National Heart, Lung, and Blood Institute or the National Institutes of Health.

COPD Foundation Funding

COPDGene is also supported by the COPD Foundation through contributions made to an Industry Advisory Board comprised of AstraZeneca, Boehringer-Ingelheim, Genentech, GlaxoSmithKline, Novartis, Pfizer, Siemens, and Sunovion.

COPDGene® Investigators – Core Units

Administrative Center: James D. Crapo, MD (PI); Edwin K. Silverman, MD, PhD (PI); Barry J. Make, MD; Elizabeth A. Regan, MD, PhD

Genetic Analysis Center: Terri Beaty, PhD; Ferdouse Begum, PhD; Peter J. Castaldi, MD, MSc; Michael Cho, MD; Dawn L. DeMeo, MD, MPH; Adel R. Boueiz, MD; Marilyn G. Foreman, MD, MS; Eitan Halper-Stromberg; Lystra P. Hayden, MD, MMSc; Craig P. Hersh, MD, MPH; Jacqueline Hetmanski, MS, MPH; Brian D. Hobbs, MD; John E. Hokanson, MPH, PhD; Nan Laird, PhD; Christoph Lange, PhD; Sharon M. Lutz, PhD; Merry-Lynn McDonald, PhD; Margaret M. Parker, PhD; Dmitry Prokopenko, Ph.D; Dandi Qiao, PhD; Elizabeth A. Regan, MD, PhD; Phuwanat Sakornsakolpat, MD; Edwin K. Silverman, MD, PhD; Emily S. Wan, MD; Sungho Won, PhD

Imaging Center: Juan Pablo Centeno; Jean-Paul Charbonnier, PhD; Harvey O. Coxson, PhD; Craig J. Galban, PhD; MeiLan K. Han, MD, MS; Eric A. Hoffman, Stephen Humphries, PhD; Francine L. Jacobson, MD, MPH; Philip F. Judy, PhD; Ella A. Kazerooni, MD; Alex Kluiber; David A. Lynch, MB; Pietro Nardelli, PhD; John D. Newell, Jr., MD; Aleena Notary; Andrea Oh, MD; Elizabeth A. Regan, MD, PhD; James C. Ross, PhD; Raul San Jose Estepar, PhD; Joyce Schroeder, MD; Jered Sieren; Berend C. Stoel, PhD; Juerg Tschirren, PhD; Edwin Van Beek, MD, PhD; Bram van Ginneken, PhD; Eva van Rikxoort, PhD; Gonzalo Vegas SanchezFerrero, PhD; Lucas Veitel; George R. Washko, MD; Carla G. Wilson, MS;

PFT QA Center, *Salt Lake City, UT*: Robert Jensen, PhD

Data Coordinating Center and Biostatistics, National Jewish Health, Denver, CO: Douglas Everett, PhD; Jim Crooks, PhD; Katherine Pratte, PhD; Matt Strand, PhD; Carla G. Wilson, MS

Epidemiology Core, University of Colorado Anschutz Medical Campus, Aurora, CO: John E. Hokanson, MPH, PhD; Gregory Kinney, MPH, PhD; Sharon M. Lutz, PhD; Kendra A. Young, PhD

Mortality Adjudication Core: Surya P. Bhatt, MD; Jessica Bon, MD; Alejandro A. Diaz, MD, MPH; MeiLan K. Han, MD, MS; Barry Make, MD; Susan Murray, ScD; Elizabeth Regan, MD; Xavier Soler, MD; Carla G. Wilson, MS

Biomarker Core: Russell P. Bowler, MD, PhD; Katerina Kechris, PhD; Farnoush BanaeiKashani, Ph.D

COPDGene® Investigators – Clinical Centers

Ann Arbor VA: Jeffrey L. Curtis, MD; Perry G. Pernicano, MD

Baylor College of Medicine, Houston, TX: Nicola Hanania, MD, MS; Mustafa Atik, MD; Aladin Boriek, PhD; Kalpatha Guntupalli, MD; Elizabeth Guy, MD; Amit Parulekar, MD;

Brigham and Women's Hospital, Boston, MA: Dawn L. DeMeo, MD, MPH; Alejandro A. Diaz, MD, MPH; Lystra P. Hayden, MD; Brian D. Hobbs, MD; Craig Hersh, MD, MPH; Francine L. Jacobson, MD, MPH; George Washko, MD

Columbia University, New York, NY: R. Graham Barr, MD, DrPH; John Austin, MD; Belinda D'Souza, MD; Byron Thomashow, MD

Duke University Medical Center, Durham, NC: Neil MacIntyre, Jr., MD; H. Page McAdams, MD; Lacey Washington, MD

Grady Memorial Hospital, Atlanta, GA: Eric Flenaugh, MD; Silanth Terpenning, MD

HealthPartners Research Institute, Minneapolis, MN: Charlene McEvoy, MD, MPH; Joseph Tashjian, MD

Johns Hopkins University, Baltimore, MD: Robert Wise, MD; Robert Brown, MD; Nadia N. Hansel, MD, MPH; Karen Horton, MD; Allison Lambert, MD, MHS; Nirupama Putcha, MD, MHS

Lundquist Institute for Biomedical Innovation at Harbor UCLA Medical Center, Torrance, CA: Richard Casaburi, PhD, MD; Alessandra Adami, PhD; Matthew Budoff, MD; Hans Fischer, MD; Janos Porszasz, MD, PhD; Harry Rossiter, PhD; William Stringer, MD

Michael E. DeBakey VAMC, Houston, TX: Amir Sharafkhaneh, MD, PhD; Charlie Lan, DO

Minneapolis VA: Christine Wendt, MD; Brian Bell, MD; Ken M. Kunisaki, MD, MS

National Jewish Health, Denver, CO: Russell Bowler, MD, PhD; David A. Lynch, MB

Reliant Medical Group, Worcester, MA: Richard Rosiello, MD; David Pace, MD

Temple University, Philadelphia, PA: Gerard Criner, MD; David Ciccolella, MD; Francis Cordova, MD; Chandra Dass, MD; Gilbert D'Alonzo, DO; Parag Desai, MD; Michael Jacobs, PharmD; Steven Kelsen, MD, PhD; Victor Kim, MD; A. James Mamary, MD; Nathaniel Marchetti, DO; Aditi Satti, MD; Kartik Shenoy, MD; Robert M. Steiner, MD; Alex Swift, MD; Irene Swift, MD; Maria Elena Vega-Sanchez, MD

University of Alabama, Birmingham, AL: Mark Dransfield, MD; William Bailey, MD; Surya P. Bhatt, MD; Anand Iyer, MD; Hrudaya Nath, MD; J. Michael Wells, MD

University of California, San Diego, CA: Douglas Conrad, MD; Xavier Soler, MD, PhD; Andrew Yen, MD

University of Iowa, Iowa City, IA: Alejandro P. Comellas, MD; Karin F. Hoth, PhD; John Newell, Jr., MD; Brad Thompson, MD

University of Michigan, Ann Arbor, MI: MeiLan K. Han, MD MS; Ella Kazerooni, MD MS; Wassim Labaki, MD MS; Craig Galban, PhD; Dharshan Vummidi, MD

University of Minnesota, Minneapolis, MN: Joanne Billings, MD; Abbie Begnaud, MD; Tadashi Allen, MD

University of Pittsburgh, Pittsburgh, PA: Frank Sciurba, MD; Jessica Bon, MD; Divay Chandra, MD, MSc; Carl Fuhrman, MD; Joel Weissfeld, MD, MPH

University of Texas Health, San Antonio, San Antonio, TX: Antonio Anzueto, MD; Sandra Adams, MD; Diego Maselli-Caceres, MD; Mario E. Ruiz, MD; Harjinder Singh
